# Copolymerization of Norbornene and Norbornadiene Using a *cis*-Selective Bimetallic W-Based Catalytic System

**DOI:** 10.3390/polym9040141

**Published:** 2017-04-18

**Authors:** Grigorios Raptopoulos, Katerina Kyriakou, Gregor Mali, Alice Scarpellini, George C. Anyfantis, Thomas Mavromoustakos, Marinos Pitsikalis, Patrina Paraskevopoulou

**Affiliations:** 1 Inorganic Chemistry Laboratory, Department of Chemistry, National and Kapodistrian University of Athens, Panepistimiopolis Zografou, Athens 15771, Greece; grigorisrap@chem.uoa.gr (G.R.); kyrkaterina@gmail.com (K.K.); 2 National Institute of Chemistry, Ljubljana 1000, Slovenia; gregor.mali@ki.si; 3 Istituto Italiano di Technologia, 16163 Genova, Italy; alice.scarpellini@iit.it (A.S.); gc.anyfantis@gmail.com (G.C.A.); 4 Organic Chemistry Laboratory, Department of Chemistry, National and Kapodistrian University of Athens, Panepistimiopolis Zografou, Athens 15771, Greece; tmavrom@chem.uoa.gr; 5 Industrial Chemistry Laboratory, Department of Chemistry, National and Kapodistrian University of Athens, Panepistimiopolis Zografou, Athens 15771, Greece; pitsikalis@chem.uoa.gr

**Keywords:** copolymerization, metathesis, norbornene, norbornadiene, ROMP, metal-metal bonds, star polymers, tungsten, viscometry

## Abstract

The bimetallic cluster Na[W_2_(*μ*-Cl)_3_Cl_4_(THF)_2_]·(THF)_3_ ({W_2_}, {W^ 3 ^W}^6+^, a′^2^e′^4^), which features a triple metal-metal bond, is a highly efficient room-temperature initiator for ring opening metathesis polymerization (ROMP) of norbornene (NBE) and norbornadiene (NBD), providing high-*cis* polymers. In this work, {W_2_} was used for the copolymerization of the aforementioned monomers, yielding statistical poly(norbornene)/poly(norbornadiene) PNBE/PNBD copolymers of high molecular weight and high-*cis* content. The composition of the polymer chain was estimated by ^13^C CPMAS NMR data and it was found that the ratio of PNBE/PNBD segments in the polymer chain was relative to the monomer molar ratio in the reaction mixture. The thermal properties of all copolymers were similar, resembled the properties of PNBD homopolymer and indicated a high degree of cross-linking. The morphology of all materials in this study was smooth and non-porous; copolymers with higher PNBE content featured a corrugated morphology. Glass transition temperatures were lower for the copolymers than for the homopolymers, providing a strong indication that those materials featured a branched-shaped structure. This conclusion was further supported by viscosity measurements of copolymers solutions in THF. The molecular structure of those materials can be controlled, potentially leading to well-defined star polymers via the “core-first” synthesis method. Therefore, {W_2_} is not only a cost-efficient, practical, highly active, and *cis*-stereoselective ROMP-initiator, but it can also be used for the synthesis of more complex macromolecular structures.

## 1. Introduction

The ring opening metathesis polymerization (ROMP) reaction belongs to the big family of metathesis, which induces the cleavage/formation of carbon–carbon double and triple bonds, allowing for the one-step synthesis of complex functional molecules. ROMP provides polyalkenamers [1,2,3] and it is considered as one of the most important tools in polymer and materials science.

Because of the properties and the applications of the unsaturated polymer materials, ROMP has been extensively studied and numerous catalytic systems have been developed. Those are mostly based on mononuclear complexes of various transition metals [4,5], which can be catalytically active per se, or after activation by a co-initiator. In any case, the active catalytic species for metathesis polymerization reactions is a metal carbene, which is either generated in situ, in which case its exact composition may remain unknown (ill-defined catalytic systems), or it has been previously synthesized and isolated, such as the Katz (W-based) [6,7,8], Schrock (Mo- or W-based) [9], and Grubbs (Ru-based) [10] alkylidenes and their numerous variations (well-defined catalytic systems). Apart from homogeneous systems, immobilized (on polymeric or inorganic support) and recyclable catalysts have also been developed [11,12].

The use of bimetallic complexes with metal-metal bonds is less common [13,14], although they provide more precise control over stereoselectivity, since both metal centers can be involved in the reaction. We have previously reported that ditungsten clusters Na[W_2_(*μ*-Cl)_3_Cl_4_(THF)_2_]·(THF)_3_ ({W_2_}, {W^ 3 ^W}^6+^, a′^2^e′^4^) and (Ph_4_P)_2_[W_2_(*μ*-Br)_3_Br_6_] ({W^ 2.5 ^W}^7+^, a′^2^e′^3^) are highly efficient and stereoselective room temperature homogeneous and/or heterogeneous initiators for the metathesis polymerization of alkynes [15,16] and the ROMP of norbornene derivatives [16,17,18,19].

The properties of ROMP-derived polymers sensitively depend on their microstructure (i.e., the *cis*/*trans* ratio of the carbon-carbon double bonds), as determined by the stereoselectivity of the reaction. Therefore, the choice of catalytic system for each reaction is important, where high activity and selectivity need to be combined with functional group tolerance. Tuning the conformation of polymers has been a long-standing problem [20,21]. W-based catalytic systems usually favor formation of polymers with higher *cis* content [16,17,18,22,23,24,25,26], while Ru-based catalytic systems show the opposite trend [27,28,29,30], with notable exceptions though [31,32]. Interestingly, {W_2_}-based catalytic systems provide high-*cis* polymers [17,18,19].

In view of the properties of catalytic system {W_2_}, we used {W_2_} for the copolymerization of norbornene (NBE) and norbornadiene (NBD). NBD bears two double bonds and usually provides insoluble cross-linked ROMP-polymers. Copolymerization of NBE and NBD could yield products with desired properties usually observed in cross-linked polymers (thermal stability, mechanical strength, etc.), while being soluble and thus more easily processable than poly(norbornadiene) (PNBD) homopolymer. In this work, we present the results of NBE and NBD copolymerization using {W_2_} as ROMP-initiator and the characterization of the products.

## 2. Materials and Methods

### 2.1. General

Starting materials were purchased from Alfa Aesar (Karlsruhe, Germany) and are of the highest available purities. Na[W_2_(*μ*-Cl)_3_Cl_4_(THF)_2_]·(THF)_3_ ({W_2_}) was prepared according to literature procedures [33]. Norbornene (NBE) was dissolved in methylene chloride, was dried over CaH_2_ under argon for 2 h and was distilled under vacuum prior to use. Norbornadiene (NBD) was passed through an Al_2_O_3_ column. All solvents were distilled under an inert atmosphere and were degassed via three freeze-pump-thaw cycles, with the exception of methanol, which was degassed with bubbling argon for 0.5 h. All operations were performed under anaerobic conditions (dinitrogen or argon atmosphere) using Schlenk techniques on an inert gas/vacuum manifold or in a dry-box (O_2_, H_2_O < 1 ppm).

Liquid NMR spectra were recorded on a Varian Unity Plus 300 spectrometer (Varian Associates Inc., Palo Alto, CA, USA). In all cases, chemical shifts are reported in ppm relative to the deuterated solvent resonances.

Solid-state NMR spectra were obtained with a 600 MHz Varian spectrometer (Palo Alto, CA, USA) operating at 150.80 MHz for ^13^C. For ^1^H-^13^C ramped CPMAS (Cross-Polarization Magic Angle Spinning) spectra, the spinning rate used was 5 kHz and the temperature for running the experiment was 25 °C.

FT-Raman spectra were obtained on a Bruker RFS-100 instrument (Bruker Optik GmbH, Lübeck, Germany) employing for excitation ca. 300 mW of the Nd:YAG 1064 nm line in a backscattering geometry. The spectra have been measured at a resolution of 4 cm^−1^ and represent averages of ca. 5000–8000 scans.

Size exclusion chromatography (SEC) experiments were carried out with a modular instrument consisting of a Waters model 600 pump (Waters Corporation, Milford, MA, USA), a Waters model U6K sample injector (Waters Corporation, Milford, MA, USA), a Waters model 410 differential refractometer (Waters Corporation, Milford, MA, USA) and a set of 4 *μ*-Styragel columns with a continuous porosity range of 10^6^–10^3^ Å. The columns were housed in an oven thermostated at 40 °C. THF was the carrier solvent at a flow rate of 1 mL/min. The instrument was calibrated with polystyrene standards covering the molecular weight range of 4000–900,000.

The thermal stability of the polymers was studied by thermogravimetric analysis (TGA) employing a Q50 TGA model from TA instruments (TA Instruments-Waters LLC, New Castle, DE, USA). Samples were placed in platinum crucibles. An empty platinum crucible was used as a reference. Samples were heated from ambient temperatures to 600 °C in a 60 mL/min flow of N_2_ at a heating rate of 10 °C/min.

The glass-transition temperatures (*T*_g_) were obtained by differential scanning calorimetry (DSC) using a 2910 modulated DSC model from TA instruments (TA Instruments-Waters LLC, New Castle, DE, USA). The samples were heated or cooled at a rate of 10 °C/min. The second heating results were obtained in all cases.

Viscometric data were analyzed using the Huggins Equation (1) and the Kraemer Equation (2), where *η*_r_, *η*_sp_ and [*η*] are the relative, specific and intrinsic viscosities, respectively, and *K_H_* and *K_K_* the Huggins and Kraemer constants, respectively. All measurements were carried out at 25 °C. Cannon-Ubbelohde dilution viscometers equipped with a Schott-Geräte AVS 410 automatic flow timer (Schott–Geräte, Hofheim, Germany) were used.

(1) $$\frac{\eta_{sp}}{c}=\left[\eta \right]+K_{H}{\left[\eta \right]}^{2}c+...$$

(2) $$\frac{\ln\eta_{r}}{c}=\left[\eta \right]-K_{K}{\left[\eta \right]}^{2}c+...$$

Scanning electron microscopy (SEM) images were taken using a field emission scanning electron microscope coupled with energy dispersive X-ray microanalysis (JEOL, JSM-7500F, Tokyo, Japan). The samples were fixed on aluminium stubs and coated with a 10 nm thick gold layer with an Emitech K950X (Quorum Technologies Ltd., East Sussex, UK). The measurements were obtained in high vacuum with a pressure of 9.6 × 10^−^^5^ Pa by using a secondary electron imaging (SEI) detector (JEOL, JSM-7500F, Tokyo, Japan) at a tension of 5 kV.

### 2.2. Catalytic Experiments

Formulations, reaction times and yields for all reactions, and molecular characteristics of all products are shown on Table 1, Table 4 and Table 5. In a typical experiment, {W_2_} (20.0 mg, 0.020 mmol) and CH_2_Cl_2_ were added to a Schlenk flask, followed by the monomers (NBE, NBD, or both). The mixture was left under stirring for a given time. After the end of the reaction, the mixture (if not gelled) was concentrated to approximately half the initial volume and a large excess of MeOH was added to precipitate the copolymer formed. The resulting solids were filtered and washed with methanol. Copolymers were characterized with ^1^H-NMR spectroscopy and SEC (the soluble part) and ^13^C CP-MAS NMR spectroscopy, TGA and DSC, and their morphology was studied with SEM. Copolymers were labelled as PNBE/PNBD xxx/xxx, whereas xxx/xxx represents the molar ratio of NBE and NBD monomers in the reaction mixture.

## 3. Results and Discussion

### 3.1. Catalyst and Catalytic Reactions

{W_2_} features a triple metal–metal bond ({W^ 3 ^W}^6+^, a′^2^e′^4^) and a face-sharing bioctahedral (FSBO) geometry. The presence of two THF ligands (one to each tungsten atom) in a *cis* arrangement to the W–W axis [33] is the key for the reactivity of this cluster towards metathesis polymerization [15]. Such species in solution, in the presence of donor ligands or coordinating solvents, may exist in equilibrium with the edge-sharing biooctahedral (ESBO) {W_2_}′ (Scheme S3) [34]. In solution, {W_2_} is air-sensitive, but in the solid state, it is stable in air at room temperature for 1–2 h. It is synthesized and recrystallized from THF and checked carefully for purity (UV-VIS) before use.

**Scheme 1:**
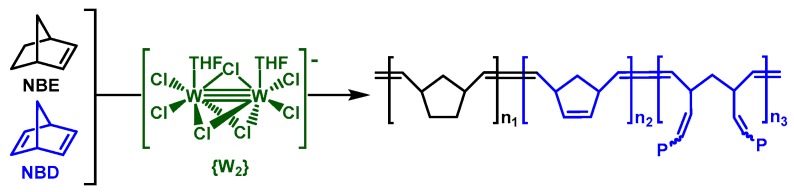
Copolymerization of NBE and NBD via ROMP with {W_2_}.

The homopolymerization of NBE and NBD (Schemes S1 and S2) with {W_2_} has been studied in detail [17,18]. Possible reaction products are shown on Schemes S1 and S2. For both monomers, the reactions were fast and quantitative in a number of solvents, among which CH_2_Cl_2_ appears to be the optimum, as it comprises low reaction times with high molecular weights and low molecular weight distributions (for PNBE; PNBD is insoluble because of its cross-linked structure). Therefore, the copolymerization of NBE and NBD with {W_2_} was studied in CH_2_Cl_2_ using different molar ratios of the two monomers, keeping the {W_2_}/monomers molar ratio at two values: (a) 1/500, which was previously found to be the optimum molar ratio for the homopolymerization of each monomer [17], and (b) 1/1400, at which ratio we would expect higher reaction rates of NBE, and therefore higher PNBE-content in the copolymer. The results are summarized in Table 1. The structure of the copolymers is shown on Scheme 1. At higher NBD/NBE molar ratios reactions were faster, which was expected, since we have previously shown that NBD polymerization with {W_2_} proceeds faster than NBE polymerization [17]. In most cases, products were insoluble, owing to the fact that NBD forms cross-linked polymers (Scheme S2). The formation of cross-linked PNBD caused reaction mixtures to gel, even at low yields, thus terminating the reactions before completion. Thus, the reaction time (*t*) shown on Table 1 is the time that the reaction stopped due to gelation. Copolymers were partially soluble in common organic solvents (CHCl_3_, CH_2_Cl_2_, THF) only when the amount of NBE used was higher than the amount of NBD. The soluble part of those copolymers was characterized with ^1^H-NMR spectroscopy and SEC. That part proved to be linear PNBE/PNBD copolymer (see below) and it had significantly lower molecular weights and broader molecular weight distributions than PNBE homopolymer. The soluble copolymer was a small fraction of the products (10–15%).

### 3.2. Chemical Characterization

**Figure 1:**
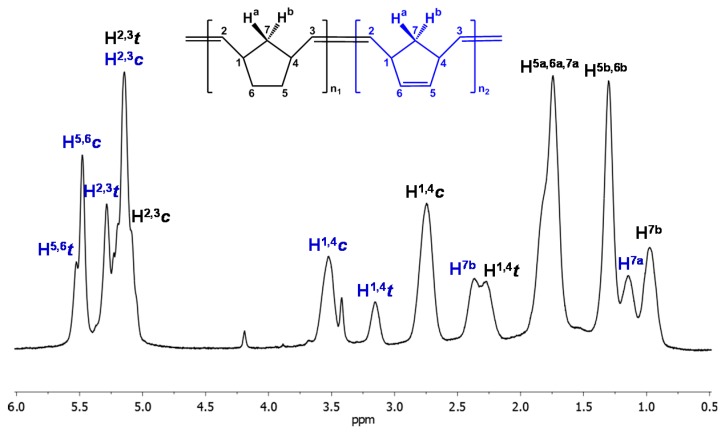
^1^H-NMR spectrum of linear PNBE/PNBD 400/100 copolymer.

Copolymers with high PNBD content were insoluble in common organic solvents. Copolymers with high PNBE content were partially soluble in CHCl_3_, CH_2_Cl_2_ and THF. The ^1^H-NMR spectra of the soluble copolymers and the assignment of the peaks are shown in Figure 1. Peaks at 5.48 and 5.53 ppm, attributed to the olefinic protons of the cyclopentene ring, clearly showed the presence of linear segments of PNBD in the polymer chain. From those spectra it is also evident that the *cis* configuration prevails, although the exact *cis*/*trans* ratio cannot be determined, due to overlapping peaks. This is in agreement with our previous findings about the stereoselectivity of {W_2_}, i.e., 86% *cis* PNBE in CH_2_Cl_2_ [17].

**Figure 2:**
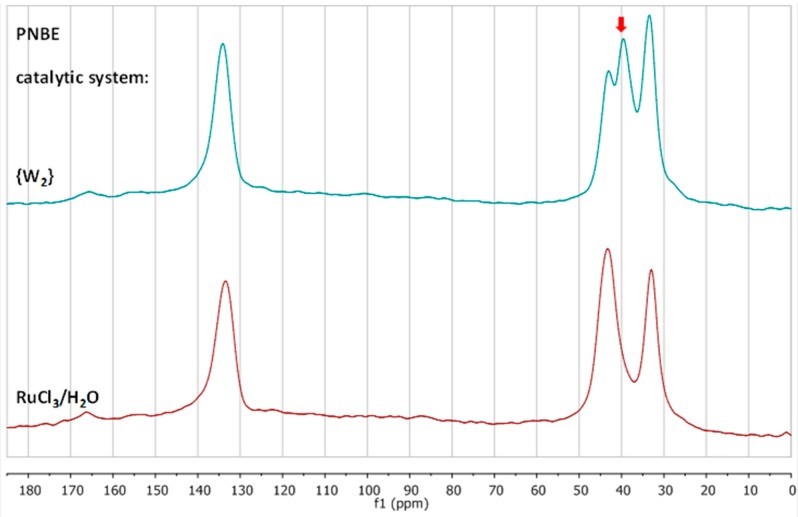
^13^C CPMAS NMR spectra of PNBE obtained with catalytic systems {W_2_} and RuCl_3_/H_2_O, as indicated. The peak assigned to *cis* segments of the polymer chain is indicated with an arrow.

Figures S1–S7 show the ^13^C CPMAS NMR spectra of all homo- and copolymers obtained in this study. For PNBE (Figure S1) olefinic carbons appear at 134.3 ppm, and aliphatic carbons at 43.0, 39.6 and 33.4 ppm, with those at 43.0 and 39.6 ppm being characteristic of the *trans* and *cis*, respectively, configuration of the polymer chain. The assignment of those peaks to *trans* or *cis* structures is based on ^13^C-NMR spectra of PNBE from the literature [35] and on the comparison of the ^13^C CPMAS NMR spectra of PNBE obtained with the catalytic systems {W_2_} and RuCl_3_/H_2_O (Figure 2). The catalytic system RuCl_3_/H_2_O is known to provide *trans*-PNBE [36]. From Figure 2, it is obvious that the peak at 39.6 ppm (assigned to the *cis* configuration) is absent from the spectrum of the Ru-generated PNBE.

For PNBD (Figure S2) olefinic carbons appear at 135.3 ppm, and aliphatic carbons at 50.3, 43.9 and 39.3 ppm, with those at 50.3 and 43.9 ppm being characteristic of the *trans* and *cis*, respectively, configuration of the polymer chain [37]. From the spectrum of PNBD the olefin coupling contribution to the formation of the cross-linked network (Scheme S2) can be determined. Cross-linking can occur either via metathesis or via olefin coupling reactions on the double bond of the cyclopentene moiety. The ratio of olefinic/aliphatic carbons depends on the mechanism of cross-linking and is equal to 4/3 (metathesis) and 2/5 (olefin coupling). Therefore, the olefin coupling contribution can be calculated via Equation (3), which takes the ratio of the integrated areas of the corresponding ^13^C CPMAS peaks as input [16]. Thus, C_olefinic_ refers to the total *sp*^2^ carbons, C_aliphatic_ to the total *sp*^3^ carbons and *x* is the fraction of polymer double bonds that participate in cross-linking via olefin coupling. Integration of the PNBD spectrum provided an olefinic/aliphatic carbons ratio equal to 1.36 (Figure S2), showing that cross-linking via olefin coupling did not occur (*x* ≈ 0).

(3) $$\frac{4-2x}{3+2x}={\left[\frac{C_{\mathrm{olefinic}}}{C_{\mathrm{aliphatic}}}\right]}_{\exp\mathrm{erimental}}$$

**Figure 3:**
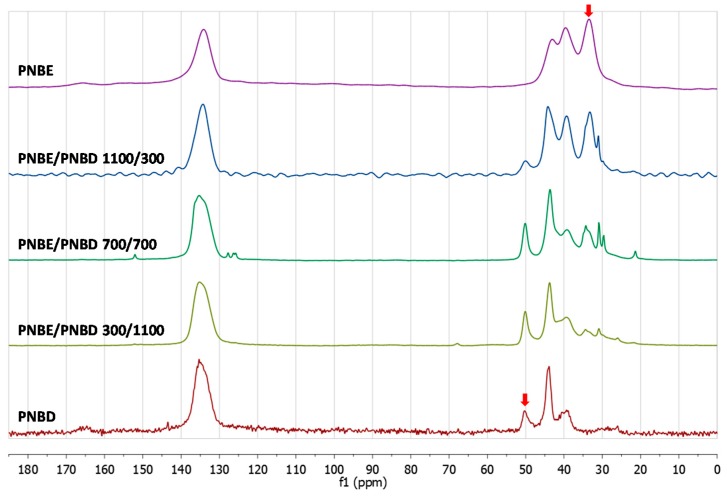
^13^C CPMAS NMR spectra of PNBE and PNBD homopolymers, and PNBE/PNBD copolymers with various NBE/NBD molar ratios, as indicated. Peaks characteristic and distinct for PNBE and PNBD are indicated with arrows.

The ^13^C CPMAS NMR spectra of all copolymers obtained in this study are similar (Figure 3). In the area where aliphatic carbons appear, the peaks corresponding to PNBE and PNBD chains overlap, which makes impossible the determination of the exact content of PNBE and PNBD segments in the polymer chain. Only qualitative conclusions can be drawn, by comparison of the relative intensity of the peaks at 33.4 and 50.3 ppm, which are characteristic of PNBE and PNBD chains, respectively. The spectra clearly show that the PNBE and PNBD content of the polymer chain depends on the molar ratio of the monomers, and increases for each monomer as the initial concentration of the monomer increases. The peak corresponding to the olefinic carbons is also very informative: it strongly resembles the analogous peak of NBE homopolymer (both appear at 134.3 ppm) for copolymers with high PNBE content; it becomes broader and shifts to 135.3 ppm, as the PNBD content increases.

Although the *cis*/*trans* content of the polymer chain cannot be accurately calculated from the ^13^C CPMAS NMR spectra, because of the overlapping PNBE and PNBD peaks, there is strong evidence that the copolymers are high-*cis*: (a) PNBE obtained with {W_2_} in CH_2_Cl_2_ (and other organic solvents) had a high *cis* content (86%) [17], and (b) the ^1^H-NMR spectra of the linear PNBE/PNBD copolymers (Figure 1) showed that the *cis* configuration prevails.

### 3.3. Thermal Properties

**Figure 4:**
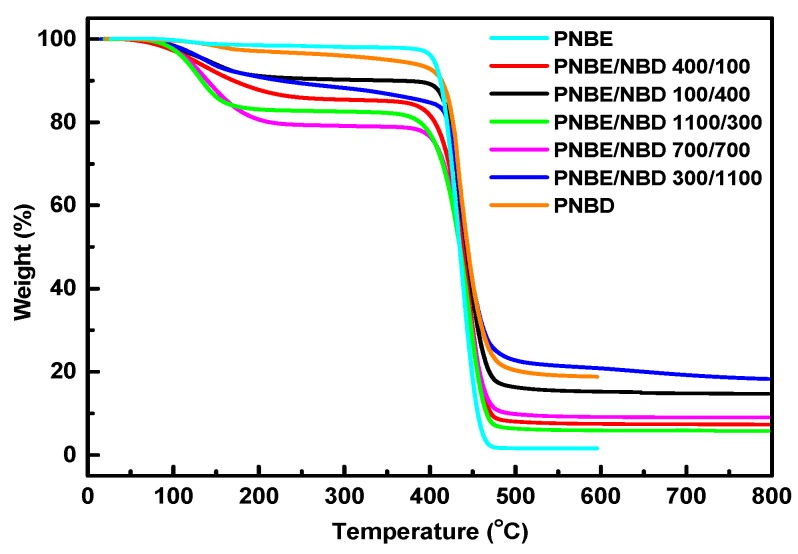
Weight loss (%) vs. temperature (°C) for all samples, as indicated.

The thermal properties of the copolymers were studied by thermogravimetric analysis (TGA) and by differential scanning calorimetry (DSC). TGA analysis results are presented in Table 2. The curves for all samples showed a continuous weight loss up to almost 500 °C, which can be divided into three stages (Figure 4). The first stage from 100 to 250 °C corresponds to evaporation and decomposition of unreacted monomers, and is more prominent for the copolymers. During the second stage, from 250 to 400 °C, the weight remained constant for all samples, except for the PNBD homopolymer and the PNBE/PNBD 300/1100 copolymer. The weight loss at this stage could be attributed to the decomposition of small polymer chains [38]. During the final stage, starting after 400 °C, all curves showed a sharp drop in the weight loss, indicating rapid degradation of the polymer backbones. It is noteworthy that TGA results provided an indication of the chemical content of the polymer chain. PNBE left practically no residue at high temperature, while PNBD homopolymer left the largest amount of residue (~20%). The residue left from the copolymers ranged from 7.4% to 18.5%, increasing as the PNBD content of the copolymers increased. That residue was characterized with FT-Raman spectroscopy. The spectrum (Figure S8) showed two characteristic peaks at 1596 and 1348 cm^−1^. From the spectrum we conclude that the residue is carbon with high *sp*^3^ content; it could be either amorphous or nanocrystalline carbon [39].

**Figure 5:**
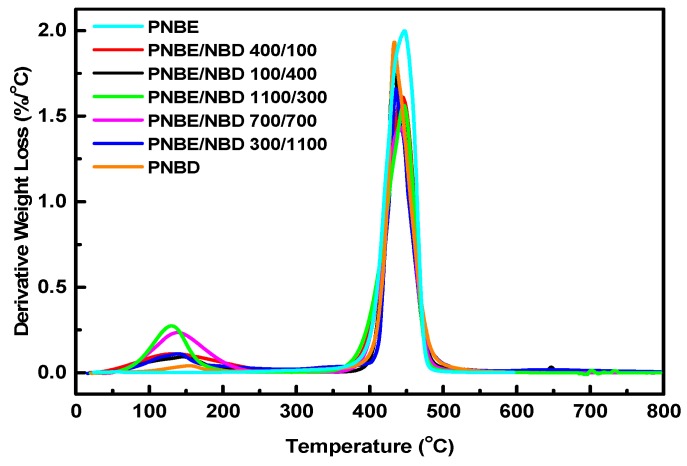
Derivative weight loss (%/°C) vs. temperature (°C) for all samples, as indicated.

Differential thermogravimetry (DTG; Figure 5) showed a single main decomposition peak for all samples, including the homopolymers (Table 2), and a smaller one at lower temperatures. This is a manifestation that the decomposition mechanism of homo- and copolymers is similar and not very complex, involving mostly reactions on the main chain. A broader decomposition temperature range was observed for PNBD compared to PNBE. On the other hand, the temperature at the peak of the decomposition temperature was lower for PNBD than for PNBE (Table 2). These two results are not contradictory and can be attributed to the structure of the different polymer chains. PNBE is a linear chain consisting of repeated ring structures, which are thermally stable. PNBD is cross-linked and contains linear chains between the cross-link points. Therefore, those linear polymer fragments decompose at lower temperatures compared to the ring structures of PNBE. This is why the temperature at the peak of the decomposition temperature is lower for PNBD than for PNBE. However, the cross-links of PNBD decompose at higher temperatures leading to broader decomposition ranges than PNBE. Therefore, the main decomposition event for both polymers is governed by the thermal stability of the macromolecular backbone, which is different for the two samples. The cross-linked structure of PNBD affects only the range of decomposition temperatures. The decomposition temperature range of the copolymers is within the values observed for the two respective homopolymers, since the copolymers combine the properties of both PNBD and PNBE homopolymers. The temperature at the decomposition peak in DTG for samples PNBE/PNBD 100/400 and 300/1100 was similar to that of the corresponding PNBD homopolymer due to their higher content in this component. On the other hand, copolymers with high PNBE contents had decomposition temperatures closer to that of PNBE homopolymer.

Differential scanning calorimetry (DSC) measurements (Table 3) showed that the glass transition temperature, *T*_g_, for PNBD was lower than that for PNBE, despite the cross-linked structure of PNBD. This result can be explained by the fact that the glass transition is attributed to the activation of segmental motions of the polymer chain. PNBD may be cross-linked, but it contains flexible linear segments between the cross-links. On the contrary, PNBE is linear but it contains rigid cyclic fragments, leading to higher *T*_g_ values compared to PNBD. DSC measurements for the copolymers (Table 3, Figures S9–S13) showed that their *T*_g_ values were lower than those of the homopolymers. This result is a strong indication that the copolymers are highly branched structures containing a large number of dangling chains. Obviously, these structures are not well defined, due to the random nature of the copolymerization reaction. The increased number of free chain ends leads to substantial reduction of the glass transition temperature according to the free volume theory [40]. Therefore, the *T*_g_ value is affected from both the macromolecular structure and the composition of the copolymer.

### 3.4. Morphology

**Figure 6:**
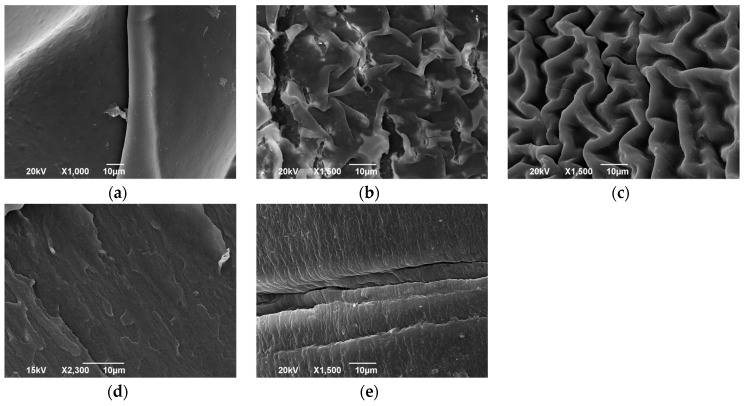
SEM images of: (**a**) PNBE homopolymer; (**b**) PNBE/PNBD 400/100 copolymer; (**c**) PNBE/PNBD 700/700 copolymer; (**d**) PNBE/PNBD 100/400 copolymer; (**e**) PNBD homopolymer.

The morphology of all materials was studied with scanning electron microscopy (SEM; Figure 6). Homopolymers exhibited smooth, non-porous morphology. Copolymers with high PNBD content featured a similar morphology, while copolymers with higher PNBE content featured a corrugated morphology. The morphology of PNBE/PNBD copolymers synthesized with {W_2_} differs significantly from the one reported by Lima-Neto et al. who used Ru-based initiators to obtain PNBE/PNBD copolymers [38,41]. In the latter case, PNBE and all copolymers were porous—in some cases, the pores had high regularity. Pore sizes and regularity decreased when PNBD content increased, with a compact surface for the copolymer obtained when the initiator/NBE/NBD molar ratio reached 1/5000/2000. This was attributed to the fact that cross-linking allowed polymer chains to approach each other, along with the fact that a higher amount of double bonds allowed polymer chains to be packed better through *π*-interactions. In our case, all materials (both the homolymers and the copolymers) were not porous. That difference could be potentially attributed to the different configuration of the polymer chains (polymers obtained using those Ru-initiators had ~50% *trans* double bond content [38,41,42], while {W_2_} provided high-*cis* polymer chains) and/or the different degree of cross-linking.

### 3.5. Dilute Solution Viscometry

The thermal properties of the copolymers provided a strong indication that those materials feature a branched-shaped structure, which behaves as a loose network. We can take advantage of the presence of the difunctional monomer NBD in order to synthesize more well-defined and soluble structures, such as multiarm star polymers. According to the literature [43,44], a living linear polymer precursor can be used as initiator for the polymerization of a small amount of a suitable difunctional monomer, leading to the formation of microgel nodules of a tightly cross-linked polymer. Those nodules serve as the branch point from which the arms of the precursor emanate. Although the functionality of the stars can be obtained directly by molecular weight determination of the arms and the star product, it is very difficult to predict and control the number of arms. The star functionality increases upon increasing the molar ratio of the difunctional monomer to the living polymer. Other parameters influencing the number of arms are the chemical nature, the concentration and the molecular weight of the living polymer chain, the temperature and the duration of the reaction, the rate of stirring etc., leading to stars with a broad distribution of functionalities. It is obvious that this method leads to rather ill-defined star polymers.

This approach can be considered as an “arm-first” method. The same technique can be applied as a “core-first” method. In the latter case, a monofunctional initiator reacts with the difunctional monomer, leading to the formation of tightly cross-linked microgel nodules bearing active sites that can be used in the next step for the polymerization of a suitable monomer. Stars of high functionality can be prepared by this method; however, the disadvantages and restrictions reported previously also apply in this case.

The “core-first” method was initially applied with the addition of a small quantity of NBD in the catalyst solution followed by the addition of NBE. The results are summarized in Table 4. For the “arm-first” method, the polymerization of NBE was initially conducted followed by the addition of a small amount of NBD. The results are presented in Table 5. In all experiments, the {W_2_}/NBE molar ratio was 1/500 and the amounts of NBD and solvent (CH_2_Cl_2_) varied. The amount of NBE was always 50–100 times higher than the amount of NBD, because otherwise insoluble copolymers were obtained.

The copolymers described in Table 4 and Table 5 were soluble in common organic solvents. In all cases, copolymers of high molecular weights (in the range of 300–800 kDa, Table 4, and 350–600 kDa, Table 5) were obtained. Moderate molecular weight distributions were obtained (1.2–1.4, Table 4, and 1.4–1.6, Table 5); the highest values were observed for copolymers synthesized via the “arm-first” method. Those results, in addition to the lower yields obtained via the “arm-first” method, show that this method is not the optimum for the synthesis of star-shaped PNBE/PNBD copolymers.

Samples 2, 3 and 8 of Table 4 were used to conduct dilute solution viscometry measurements, because those samples were obtained at the highest yield. The intrinsic viscocity, [*η*], of those copolymers was measured. The theoretical intrinsic viscosity, [*η*]_lin_, of linear polymers of the same molecular weight was calculated from Equation (4), where *M*_w_ is the molecular weight, determined with SEC. The values of *K* = 0.0701 mL/g and *α* = 0.64 are known from the literature [45] for polymers of similar structures.

(4) $${\left[\eta \right]}_{lin}=KM_{w}{}^{a}$$

Star polymers are distinguished from linear polymers by their smaller size and therefore their higher segment density. The degree of contraction depends on the functionality of the star and can be normalized compared to the size of a single arm of the star or a linear polymer having the same molecular weight as the star polymer [46]. The second case is more common in the literature.

The degree of branching can be calculated using the Zimm-Stockmayer theory and considering the star model [47]. The branching factor *g* was introduced, defined from Equation (5), where <*S*^2^>_0,br_ and <*S*^2^>_0,l_ are the radii of gyration of the branched and the corresponding linear polymer, respectively, having the same molecular weight. The subscript *th* denotes that the random-flight model was adopted. For star structures, Equations (6) and (7) have been proposed, where *f* is the functionality of the star, or else, the number of arms.

(5) $$g_{\mathrm{th}}=\frac{{\langle S^{2}\rangle }_{0,\mathrm{br}}}{{\langle S^{2}\rangle }_{0,l}}$$

(6) $$g_{\mathrm{th}}=\frac{3f-2}{f^{2}}\;\mathrm{for}\;\mathrm{regular}\;\mathrm{stars}$$

(7) $$g_{\mathrm{th}}=\frac{6f}{\left(f+1)(f+2\right)}\;\mathrm{for}\;\mathrm{stars}\;\mathrm{with}\;\mathrm{random}\;\mathrm{distribution}\;\mathrm{of}\;\mathrm{arms}$$

It is difficult to measure the radius of gyration of a polymer with accuracy. It is much easier and more accurate to measure the intrinsic viscosity by dilute solution viscometry. Therefore, another branching parameter has been proposed (Equation (8)), where, [*η*]_br_ and [*η*]_lin_ are the intrinsic viscosities of branched and linear polymers, respectively, having the same molecular weight.

(8) $$g\prime =\frac{{\left[\eta \right]}_{\mathrm{br}}}{{\left[\eta \right]}_{\mathrm{lin}}}$$

The two branching parameters *g*_th_ and *g*’ are related through the Equation (9), where the exponent *ε* is equal to 0.5 for regular stars and equal to 0.7 for stars with random distribution of arms.

(9) $$g\prime =g_{\mathrm{th}}{}^{\varepsilon }$$

Therefore, the *g*’ values of the star branched polymers were measured experimentally. Employing Equation (9), the *g*_th_ values were calculated using the random distribution of arms, since this assumption reflects more closely the structures under consideration. It was considered that the exponent *ε* was equal to 0.7 for stars with random distributions of arms. Finally, using Equations (6) and (7) and the calculated *g*_th_ values, the functionality of the star polymers was estimated for both cases.

**Figure 7:**
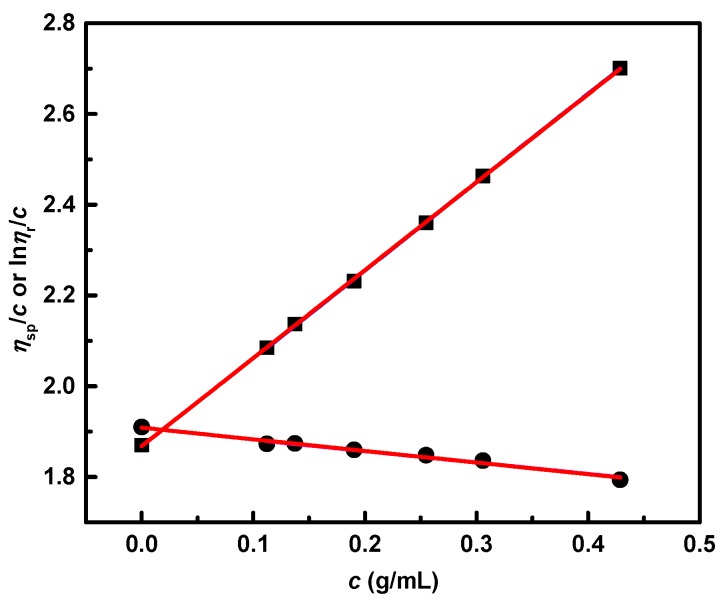
Viscometry results of sample 2 (Table 4) in THF at 35 °C.

Results are presented in Table 6 and Figure 7. For samples 2 and 8 the [*η*]/[*η*]_lin_ ratio was lower than 1, indicating that those copolymers had a more compact structure. Adopting the above-mentioned analysis, the functionality of the stars was calculated. For sample 2 the functionality was much lower than that of sample 8. This result may be attributed to the longer polymerization time used to produce sample 8. This prolonged period may lead to extended cross-linking of NBD and thus to larger cores with higher functionalities. For sample 3 the corresponding ratio was higher than 1, possibly due to a cross-linking process. For the synthesis of that sample, the lowest amount of NBD was employed and the polymerization reaction was conducted in the most dilute solution among the other samples. Therefore, the cross-linking density was not very high, meaning that double bonds of the monomer remained unreacted. Subsequent polymerization of NBE led to partial cross-linking through reactions with those pendant double bonds of the core material leading to star-star coupling. These products were still soluble, but their size was much higher than that of a simple star polymer.

In the past, several research groups have tried to prepare star polymers by ROMP reactions by adopting various methodologies. Schrock et al. [48,49] employed both *exo*-*trans*-*exo*-pentacyclo-[8.2.1.1^4,7^.0^2,9^.0^3,8^]tetradeca-5,11-diene and *endo*-*cis*-*endo*-hexacyclo-[10.2.1.1^3,10^.1^5,8^.0^2,11^.0^4,9^]heptadeca-6,13-diene as difunctional monomers either through the “arm-first” or the “core-first” methodology for the synthesis of PNBE stars using Mo-catalysts. The “core-first” approach afforded only linear PNBE chains and gels, in contrast to the results of the present work. On the other hand, the “arm-first” approach resulted in soluble products. However, SEC analysis revealed that a mixture of products with different functionalities and of broad molecular weight distribution was obtained. Dounis and Feast [50] reported the synthesis of three-arm stars through the coupling reaction of living polymer chains produced via ROMP, using well-defined W-catalysts, with trifunctional aldehydes. Disadvantages of this methodology are the facts that a good control over the stoichiometry is needed and that only stars of low functionalities can be prepared through this route. Multinuclear dendritic Ru-complexes were prepared and served as multifunctional catalysts for the synthesis of PNBE stars [51,52]. Products of broad molecular weight distribution were obtained in all cases.

The initial results of the present work revealed that there is a good possibility to control the molecular structure and avoid the presence of insoluble products, leading to well-defined star polymers. More work is in progress on this direction and the results will be presented in a forthcoming publication.

## 4. Conclusions

Statistical poly(norbornene)/poly(norbornadiene) PNBE/PNBD copolymers were synthesized via ROMP using Na[W_2_(*μ*-Cl)_3_Cl_4_(THF)_2_]·(THF)_3_ ({W_2_}) as initiator. The composition of the polymer chain was estimated, although not quantitatively, by ^13^C CPMAS NMR data, and it was found that the ratio of PNBE/PNBD segments in the polymer chain is relative to the monomer molar ratio in the reaction mixture. Solid-state NMR spectroscopy of the homopolymers revealed high-*cis* structures and that the only cross-linking mechanism operating for PNBD was the metathetic one (no radical coupling). The thermal properties of all copolymers were similar, resembled the properties of PNBD homopolymer and indicated a high degree of cross-linking for both PNBD homopolymer and the copolymers. PNBD homopolymer left ~20% residue after pyrolysis, which was shown by FT Raman to be carbon with high *sp*^3^ content; the residue left from the copolymers increased as the PNBD content of the copolymers increased. The morphology of all materials in this study (observed with SEM) was smooth and non-porous; copolymers with higher PNBE content featured a corrugated morphology. Glass transition temperatures were lower for the copolymers than for the homopolymers, providing a strong indication that these materials featured a branched-shaped structure. This conclusion was further supported by viscosity measurements of copolymers solutions in THF. Star-shaped structures could be obtained via the “core-first” synthesis method. Therefore, the molecular structure of those materials can be controlled, potentially leading to well-defined star polymers. This can be added to the unique features of {W_2_} reactivity (i.e., high activity and stereoselectivity), making {W_2_} a cost-efficient and practical ROMP-initiator for the synthesis of high-*cis* (co)polymers and more complex macromolecular structures.

## Figures and Tables

**Table 1:** Reactions of {W_2_} with NBE and NBD ^a^.

Sample	{W_2_}/ΝΒΕ/NBD (Molar Ratio)	*t* (min)	Yield (%)	*M*_w_ × 10^−3 b^	*M*_w_/*M*_n_^b^
PNBE ^c^	1/500/0	60	96	529	1.2
PNBE/PNBD 400/100	1/400/100	10	61	129	2.0
PNBE/PNBD 100/400	1/100/400	3	99	^d^	^d^
PNBE/PNBD 1100/300	1/1100/300	12	11	252	2.5
PNBE/PNBD 700/700	1/700/700	7	18	^d^	^d^
PNBE/PNBD 300/1100	1/300/1100	5	66	^d^	^d^
PNBD ^c^	1/0/500	6	>99	^d^	^d^

^a^ Conditions: {W_2_} (20.0 mg, 0.020 mmol), monomer(s), CH_2_Cl_2_ (homopolymerization, 2 mL; copolymerization, 15 mL); ^b^ By SEC in THF at 40 °C; ^c^ Results obtained from Ref. [17]; ^d^ Insoluble (co)polymer.

**Table 2:** TGA results (main decomposition peak) at the heating rate of 10 °C/min.

Sample	Start (°C)	Finish (°C)	Peak (°C)	Residue (%)
PNBE	376.11	490.49	445.96	1.60
PNBE/PNBD 400/100	353.54	495.96	443.78	7.35
PNBE/PNBD 100/400	377.78	509.09	432.86	14.75
PNBE/PNBD 1100/300	348.48	491.92	446.59	5.78
PNBE/PNBD 700/700	363.64	508.08	442.67	9.06
PNBE/PNBD 300/1100	389.90	539.39	435.51	18.46
PNBD	377.69	561.96	433.42	18.89

**Table 3:** Experimental *T*_g_ values.

Sample	*T*_g_ (°C)
PNBE	57.9
PNBE/PNBD 400/100	37.9
PNBE/PNBD 100/400	39.5
PNBE/PNBD 1100/300	39.4
PNBE/PNBD 700/700	29.1
PNBE/PNBD 300/1100	47.5
PNBD	50.1

**Table 4:** Formulations and results of the “core-first” method at {W_2_}/NBE molar ratio 1/500 in CH_2_Cl_2_^a^.

Entry	CH_2_Cl_2_ (mL)	NBD (μL)	*t*_1_^b^ (min)	*t*_2_^c^ (h)	Yield (%)	*M*_w_ × 10^−3^ ^d^	*M*_w_/*M*_n_^d^
1	15	10	10	1	9	-	-
2	15	20	5	1	22	320	1.41
3	25	10	15	21	32	429	1.42
4	25	20	5	21	16	771	1.29
5	25	10	40	6	8	764	1.30
6	25	20	40	6	3	661	1.24
7	25	10	90	30	10	423	1.37
8	25	20	90	30	35	566	1.31

^a^ Conditions: {W_2_} (20.0 mg, 0.020 mmol), NBD (10.0 μL, 9.0 mg, 98 μmol or 20.0 μL, 18.0 mg, 196 μmol), NBE (941 mg, 10.0 mmol), CH_2_Cl_2_. ^b^ Reaction time for NBD polymerization. ^c^ Reaction time for NBE polymerization. ^d^ By SEC in THF at 40 °C.

**Table 5:** Formulations and results of the “arm-first” method at {W_2_}/NBE molar ratio 1/500 in CH_2_Cl_2_^a^.

Entry	NBD (μL)	*t*_1_^b^ (min)	*t*_2_^c^ (h)	Yield (%)	*M*_w_ × 10^−3 d^	*M*_w_/*M*_n_^d^
1	10	40	30	10	430	1.41
2	20	40	30	5	436	1.60
3	10	40	30	5	352	1.53
4	20	40	30	10	564	1.52
5	10	30	30	15	463	1.56
6	100	30	30	30	390	1.42

^a^ Conditions: {W_2_} (20.0 mg, 0.020 mmol), NBD (10.0 μL, 9.0 mg, 98 μmol or 20.0 μL, 18.0 mg, 196 μmol), NBE (941 mg, 10.0 mmol), CH_2_Cl_2_ (25 mL). ^b^ Reaction time for NBD polymerization. ^c^ Reaction time for NBE polymerization. ^d^ By SEC in THF at 40 °C.

**Table 6:** Viscosity measurements of copolymers in THF solutions at 35 °C.

Sample (Table 4)	*M*_w_ × 10^−3^ ^a^	[*η*] (mL/g)	[*η*]_lin_ (mL/g)	*g’*	Functionality
2	320	186.629	233.47	0.799	4.9
3	429	529.04	282.22	1.874	
8	566	65.321	336.83	0.194	59.4

^a^ By SEC in THF.

## Supplementary Materials

The following are available online at www.mdpi.com/2073-4360/9/4/141/s1; Scheme S1: NBE homopolymerization via ROMP; Scheme S2: NBD homopolymerization via ROMP; Scheme S3: Schematic representation of the potential equilibrium between {W_2_} and {W_2_}′; Figure S1: ^13^C CPMAS NMR spectrum of PNBE homopolymer; Figure S2: ^13^C CPMAS NMR spectrum of PNBD homopolymer; Figure S3: ^13^C CPMAS NMR spectrum of PNBE/PNBD 100/400 copolymer; Figure S4: ^13^C CPMAS NMR spectrum of PNBE/PNBD 400/100 copolymer; Figure S5: ^13^C CPMAS NMR spectrum of PNBE/PNBD 1100/300 copolymer; Figure S6: ^13^C CPMAS NMR spectrum of PNBE/PNBD 700/700 copolymer; Figure S7: ^13^C CPMAS NMR spectrum of PNBE/PNBD 300/1100 copolymer; Figure S8: FT-Raman spectrum of the residue left after anaerobic heating up to 800 °C of PNBE/PNBD 100/400 copolymer; Figure S9: DSC thermograph of PNBE/PNBD 400/100 copolymer; Figure S10: DSC thermograph of PNBE/PNBD 100/400 copolymer; Figure S11: DSC thermograph of PNBE/PNBD 1100/300 copolymer; Figure S12: DSC thermograph of PNBE/PNBD 700/700 copolymer; Figure S13: DSC thermograph of PNBE/PNBD 300/1100 copolymer.
