# Supplementary material for: Copolymerization of Norbornene and Norbornadiene Using a cis-Selective Bimetallic W-Based Catalytic System
Source: Polymers (Basel). 2017 Apr 18;9(4):141. doi: 10.3390/polym9040141 (PMC6432165; doi:10.3390/polym9040141)
Supplement: Supplementary file 1 [file polymers-09-00141-s001.pdf]

Supplementary Materials: Copolymerization of Norbornene and Norbornadiene Using a *Cis*-Selective Bimetallic W-Based Catalytic System. Opening a Route to Afford Star Polymers

Grigorios Raptopoulos, Katerina Kyriakou, Gregor Mali, Alice Scarpellini, George C. Anyfantis, Thomas Mavromoustakos, Marinos Pitsikalis and Patrina Paraskevopoulou

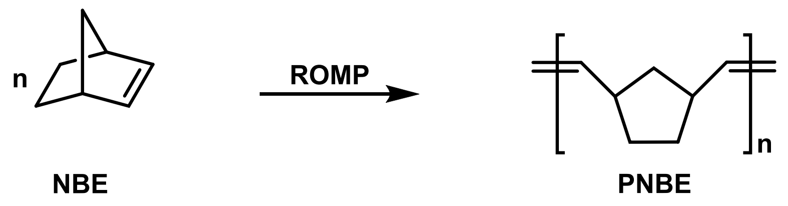

Scheme S1. NBE homopolymerization *via* ROMP.

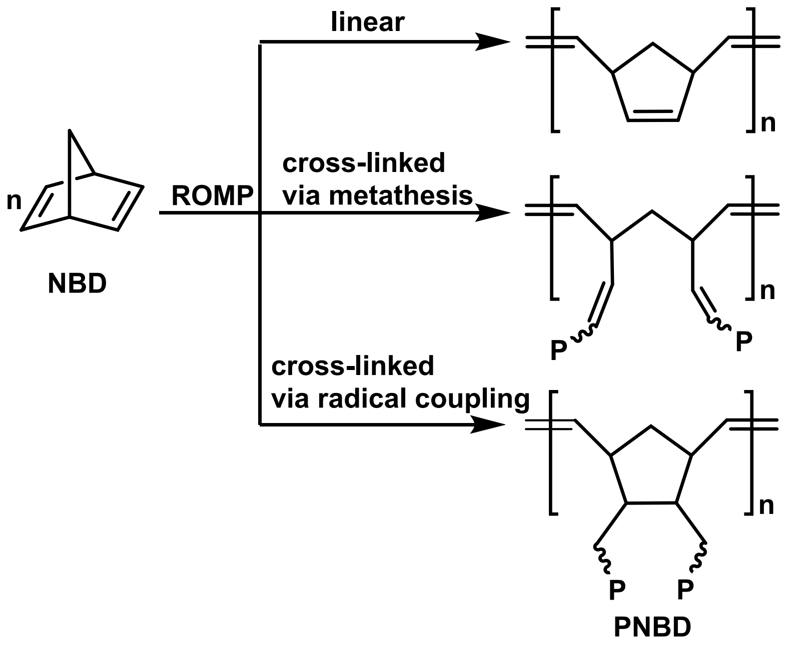

Scheme S2. NBD homopolymerization *via* ROMP.

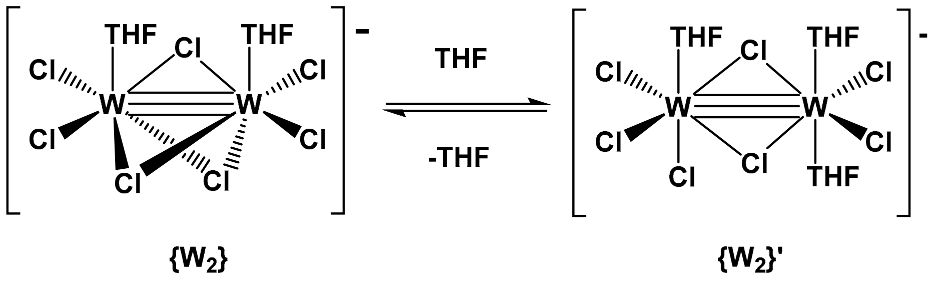

Scheme S3. Schematic representation of the potential equilibrium between **{W2}** and **{W2}'**.

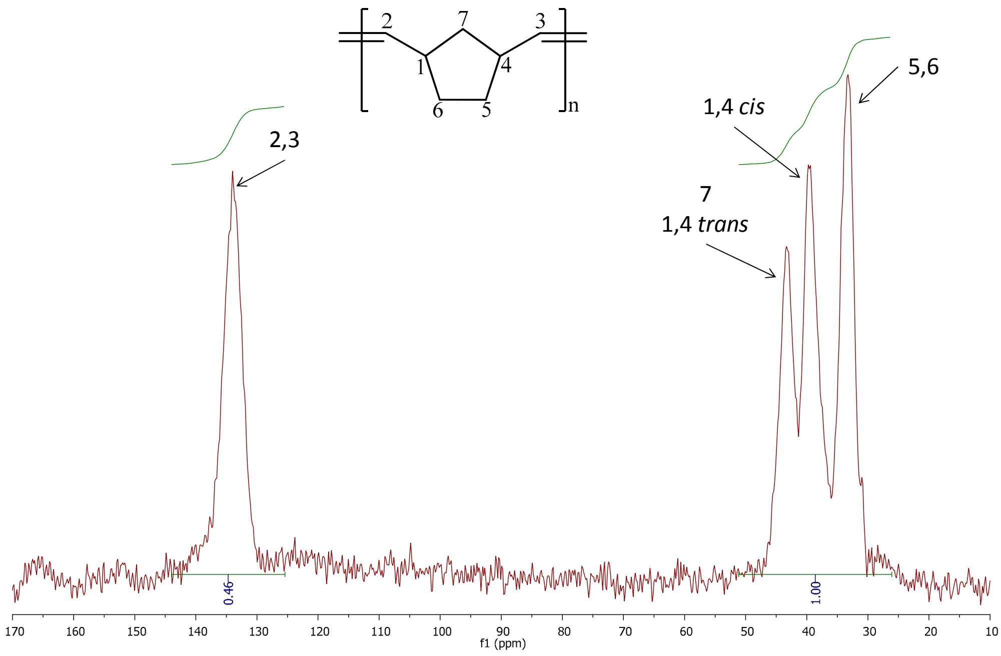

Figure S1. ^{13}C CPMAS NMR spectrum of **PNBE** homopolymer.

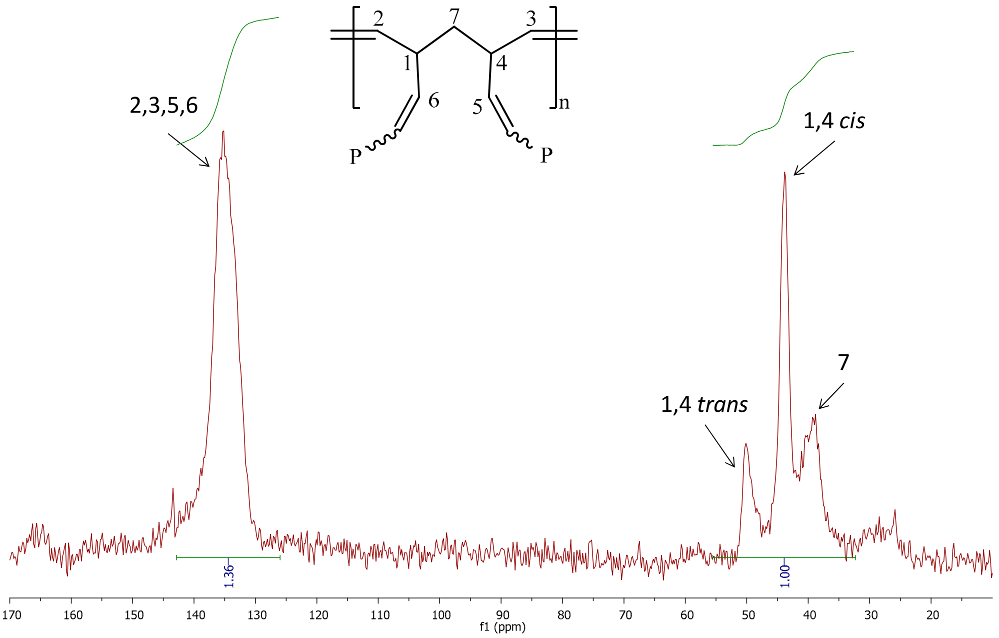

Figure S2. ^{13}C CPMAS NMR spectrum of **PNBD** homopolymer.

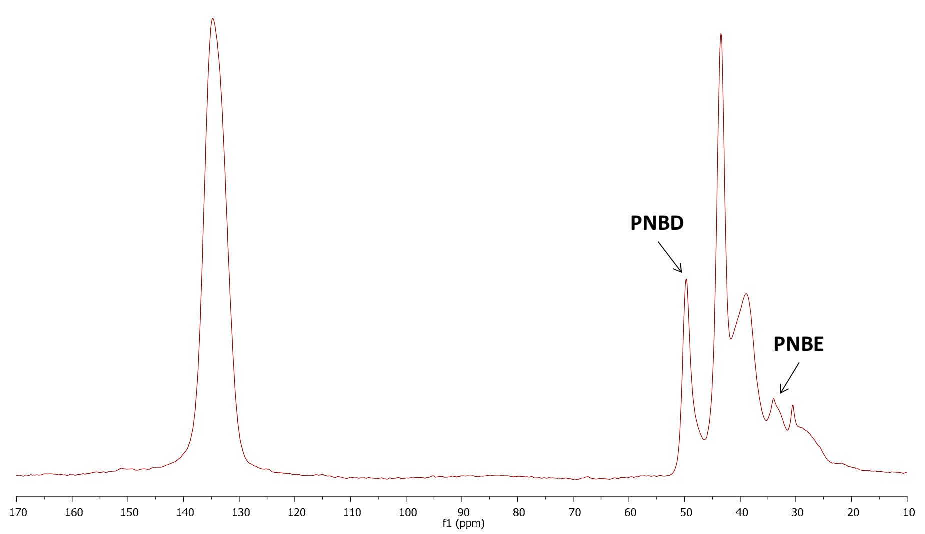

Figure S3. ^{13}C CPMAS NMR spectrum of **PNBE/PNBD 100/400** copolymer.

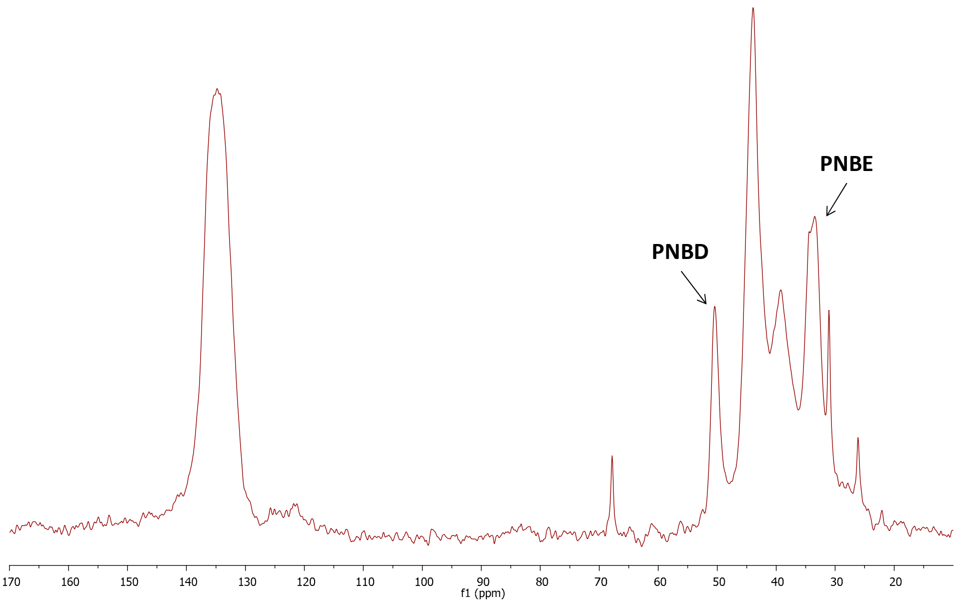

Figure S4. ^{13}C CPMAS NMR spectrum of **PNBE/PNBD 400/100** copolymer.

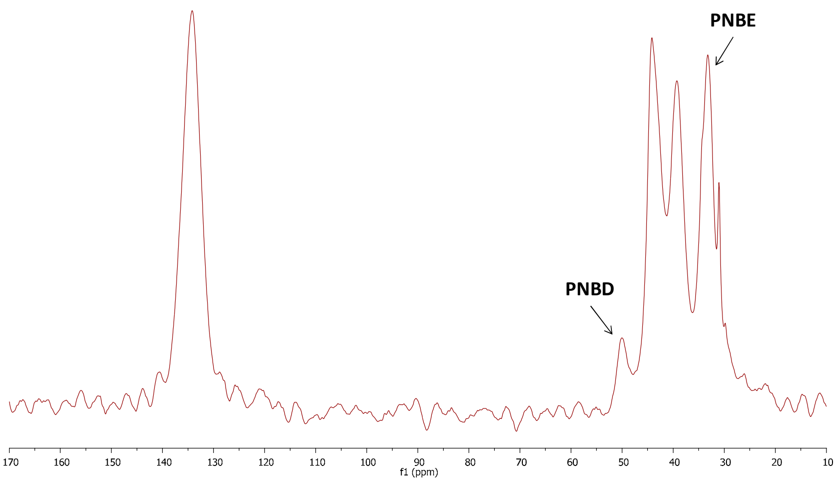

Figure S5. ^{13}C CPMAS NMR spectrum of **PNBE/PNBD 1100/300** copolymer.

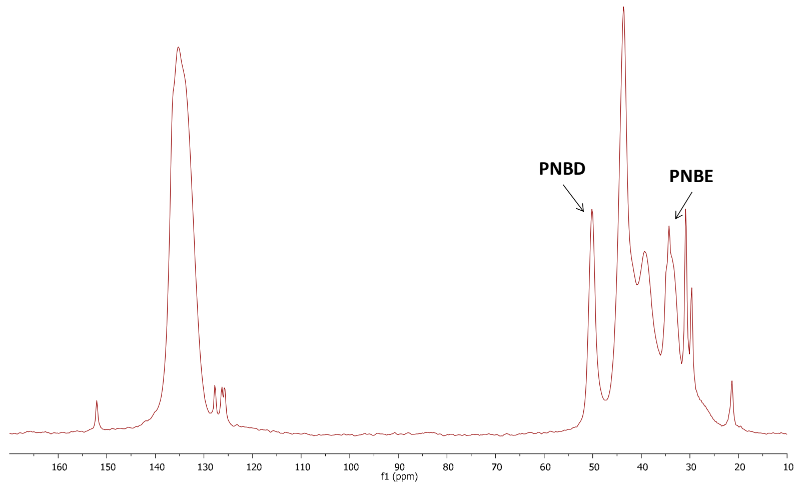

Figure S6. ^{13}C CPMAS NMR spectrum of **PNBE/PNBD 700/700** copolymer.

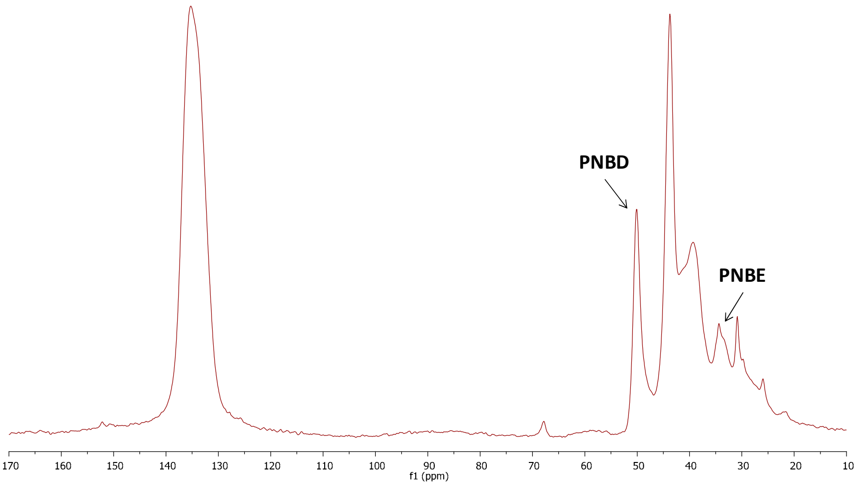

Figure S7. ^{13}C CPMAS NMR spectrum **of PNBE/PNBD 300/1100** copolymer.

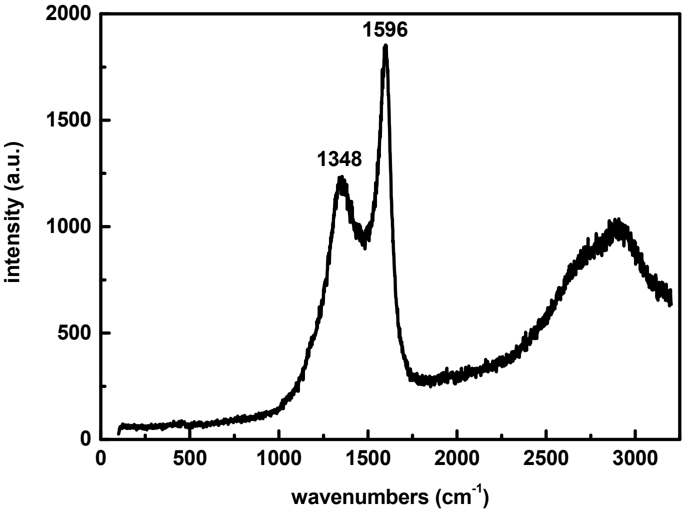

Figure S8. FT-Raman spectrum of the residue left after anaerobic heating up to 800 °C of **PNBE/PNBD 100/400** copolymer.

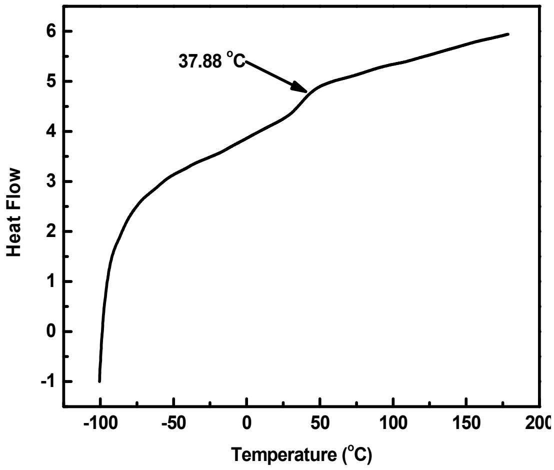

Figure S9. DSC thermograph of **PNBE/PNBD 400/100** copolymer.

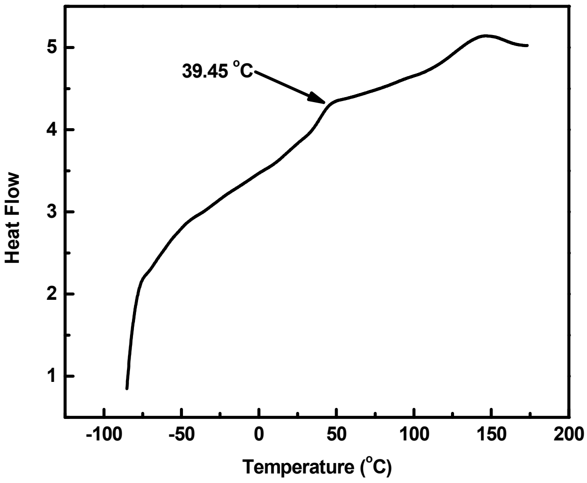

Figure S10. DSC thermograph of **PNBE/PNBD 100/400** copolymer.

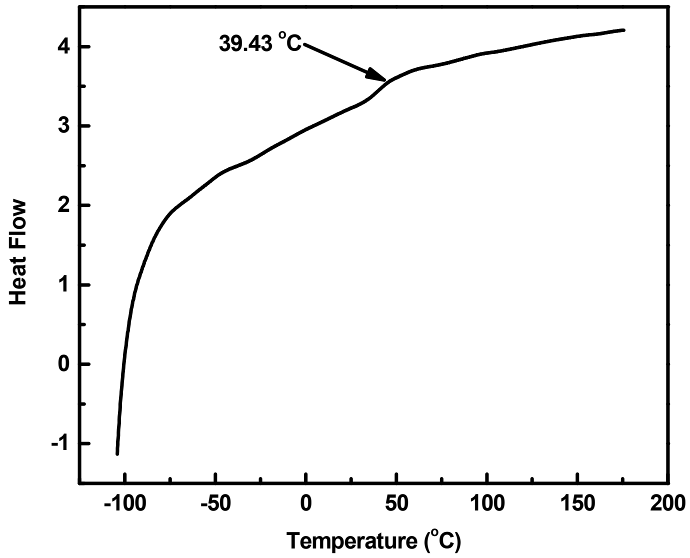

Figure S11. DSC thermograph of **PNBE/PNBD 1100/300** copolymer.

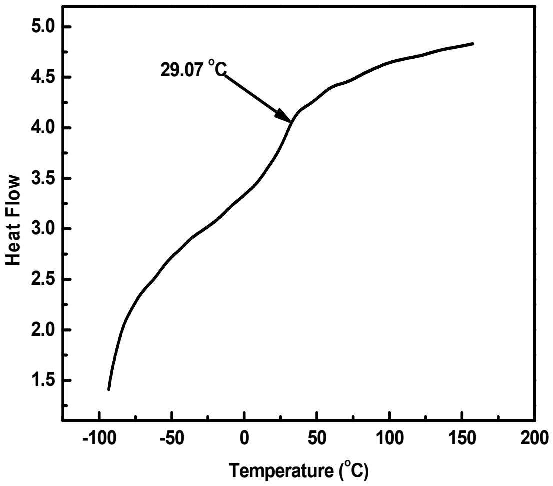

Figure S12. DSC thermograph of **PNBE/PNBD 700/700** copolymer.

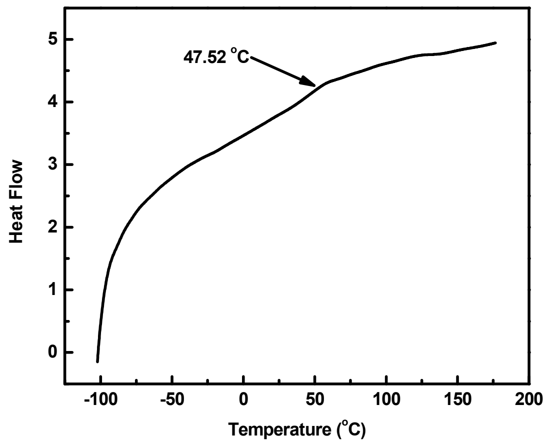

Figure S13. DSC thermograph of **PNBE/PNBD 300/1100** copolymer.
